# Annotations

**Published:** 1910-07-23

**Authors:** 


### Annotations.

### Depopulation in France.

A Bill drafted by Dr. Lannelongue, countersigned by twenty eminent Frenchmen, has been introduced into the Senate with the object of stemming the tide of depopulation in France. It is maintained by many authorities with a good show of reason that the existing law fixing very narrow limits to a father's bequest to his children, and compelling him to leave something to each relative dependent upon him, has been probably the principal influence in favour of depopulation. This law, which compelled fathers to divide the estate, if they were blessed with more than one son or daughter, has proved in practice a considerable obstacle to families with more than one child. The Bill therefore provides for the cancellation of the existing law, so that parents shall have in future full freedom to bequeath their money as they wish. The Bill further provides that every man unmarried at the age of 29 will be called upon to fulfil extra periods of military service until he has been inscribed for 25 years on the active or reserve forces. No one will be retained in a State or municipal department if not married at 25, while every civil servant who is the father of three children alive shall benefit by promotion and have in addition a bounty of £8 yearly for each child beyond the third child. Civil servants will also enjoy a supplementary pension benefit of £4 annually for each living child beyond three. It is expected that the Bill, though influentially backed, will give rise to much discussion and some determined opposition. It cannot fail to excite interest in this country, and in its favour much may be urged in view of the present depopulation of France.

### A Death under "Open Ether."

In view of the recent interest aroused among the lay public by a long article on the necessity for legislation concerning anæsthetics which has quite recently been printed in a prominent place by the most widely circulated daily newspaper in Britain, it is perhaps important that the attention of the profession should be focussed on a recent inquest on a patient deceased during an operation for appendicitis. For the laity often question medical men on such technical topics as the Press choose to ventilate, and this inquest emphasises once more the old truth that every anæsthetic is a poison, and that no anæsthetic is absolutely safe. The facts are simply that a girl of eighteen was operated upon late one night in a hospital for acute suppurative appendicitis, and that about the end of the operation respiratory and circulatory failure were observed. Despite half an hour of direct heart massage through the diaphragm, recovery was only partial and temporary, and death took place some two hours and a half after the first untoward symptoms developed. The chief point for comment is that the administration was throughout that of the open-ether method, preceded by the usual dose of mòrphine with atropine. It has been freely stated that fatalities on the table are unknown when this sequence is employed, and such statements have attained considerable circulation. It is, however, to be admitted that fatalities do occur, though they are rare; and it is as well that medical men should recognise this fact. It is also often said that respiratory complications never occur, as they certainly do in a small percentage of cases when ether is given by closed methods; but this also is an over-statement. In certain clinics on the Continent and across the Atlantic it is the fashion to entrust the anæsthetic to a nurse or other unqualified assistant of the operator; and where this is done it is easy to understand that an anæsthetic must be chosen which, despite other disadvantages, possesses the widest "margin of error." But where, as here, anæsthetisation in hospitals is entirely in the hands of qualified medical men, the personality of the administrator becomes an important factor, and it is by no means so certain that open ether is the ideal routine drug for ordinary patients.

### The Medical Uses of Mummies.

Mediæval medicine employed strange remedies, some of them fantastical but soothing, such as frog's spawn; others creepy and formidable, such as the wood of coffins. Dr. Wiedemann, Professor of Egyptology at the University of Bonn, assures us that it is not very long since medicines made from mummies were employed in the treatment of disease. The mummies made use of were of two kinds, genuine and artificial. The former were snatched by the Arabs from the burial-places of the valley of the Nile and sent into Europe and Asia. The therapeutic virtues of these were attributed to the asphalt with which the embalmer had impregnated the bodies, and of which Galen and the other Greek physicians acknowledged the healing virtues in cases of colds, eczema, convulsions, epilepsy, suppuration, and other maladies. The author also asserts that the very name—mummy—is derived from a Persian and Arabic word meaning asphalt, and that it was only at a later date that the word was used exclusively to refer to embalmed bodies. Mummies were so generally used in Persia as remedies that the Shah offered them as presents to friendly sovereigns. Louis XIV. and Catherine received gold boxes filled with mummified limbs, and as late as 1809 Queen Caroline of England was ordered mummy extract by her physicians. In the absence of authentic mummies, such as the soil of Egypt alone could furnish, an artificial variety was manufactured in other countries. Here is a somewhat disquieting receipt according to the manuscript of the Persian poet Nizami: "Take a man with red skin and hair; feed him with fruits up to the age of thirty. Then plunge him into a stone vat filled with honey and divers other drugs; close up the vat and seal it hermetically. One hundred and twenty years later the honey and body will be mummified. Open the vat and serve up the contents." The mummy extract, says the German savant, was in common use in the eighteenth century, and as late as 1853 it figured in Austrian pharmacy.

